# *Brevibacterium ravenspurgense* bacteremia

**DOI:** 10.1016/j.idcr.2025.e02318

**Published:** 2025-07-09

**Authors:** Fang Xingyan, Ou Xingkun, Hu Yonglin, Daimin Xiao

**Affiliations:** a Department of Clinical Laboratory, Kweichow Moutai Hospital, Zunyi 564500, China; b Department of Clinical Laboratory, Affiliated Hospital of Zunyi Medical University, Zunyi, Guizhou 563100, China

**Keywords:** *Brevibacterium ravenspurgense*, Bacteremia, 16S rRNA

## Abstract

*Brevibacterium ravenspurgense* infections are exceptionally rare and frequently necessitate sophisticated laboratory analyses for precise identification. In this report, we detail a case of *Brevibacterium ravenspurgense* bacteremia in a patient suffering from adrenoleukodystrophy, a rare hereditary metabolic disorder. The diagnosis was validated using 16S rRNA gene sequencing, emphasizing the pivotal role of advanced molecular methodologies in pinpointing uncommon pathogens.Vancomycin was the preferred antibiotic for the treatment of *Brevibacterium ravenspurgense* infection. This case accentuates the importance of considering rare pathogens, particularly in patients with intricate medical backgrounds.

## Introduction

*Brevibacterium*, a genus of Gram-positive, non-spore-forming, catalase-positive bacteria, consists of 50 recognized species, only ten of which have been isolated from clinical samples [1], [2]. *Brevibacterium ravenspurgense*, initially described in 2008, was isolated from wounds and human blood cultures [3]. In 2009, *B.massiliense* was first described but later identified as a heterotypic synonym of *B.ravenspurgense* [4]. Adrenoleukodystrophy (ALD) is a rare genetic disorder characterized by the accumulation of very long chain fatty acids in the brain and adrenal cortex, leading to progressive neurological deterioration and immunodeficiency. Here, we present a case of *B.ravenspurgense* bacteremia in an adult patient with ALD, which, to our knowledge, is the first reported case in China.

## Case presentation

A 45-year-old male, diagnosed with adrenoleukodystrophy six months ago and hospitalized for over three months at a local hospital, presented to our emergency department in September 2023 with a progressively worsening mental status.

Upon physical examination, the patient was found to be unconscious and malnourished, with scattered rashes visible on his body. He still had a subclavian peripherally inserted central catheter (PICC), which showed no signs of erythema, pus, or tenderness at the insertion site. Coarse rales were detected in both lungs, and moist rales were heard in the bilateral lower lung lobes. Chest CT revealed pulmonary parenchymal ground-glass opacities in the bilateral lower lung lobes, accompanied by small amounts of pleural effusion. Laboratory studies showed a white blood cell count (WBC) of 10.31 * 10^9/L (reference range, 3.5–9.5 * 10^9/L), a neutrophil count of 8.79 * 10^9/L (reference range, 1.8–6.3 * 10^9/L), and a C-reactive protein (CRP) level of 20.32 mg/L (reference range, 0.07–8.2 mg/L). The patient was empirically started on intravenous piperacillin sodium tazobactam (4.5 g q8h) for the treatment of pneumonia.

On day 14, symptoms of chills and high fever appeared, with a body temperature of 38°C, blood pressure of 102/67 mmHg, a pulse rate of 74 beats per minute, and a respiratory rate of 26 breaths per minute. The laboratory results indicated that the WBC count was 12.53 * 10^9/L, the neutrophil count was 10.64 * 10^9/L and the CRP level was 38.26 mg/L (Table 1).

**Table 1:** Indicators of different hospitalization days.

Day of hospitalization	Temperature (℃)	WBC (*10^9/L)	Neutrophil count (*10^9/L)	CRP (mg/L)
Day 1	36.5	10.31	8.79	20.32
Day 14	38.0	12.53	10.64	38.26
Day 21	37.0	6.82	4.97	15.81

WBC, white blood cell; CRP, C-reactive protein

**Fig. 1:**
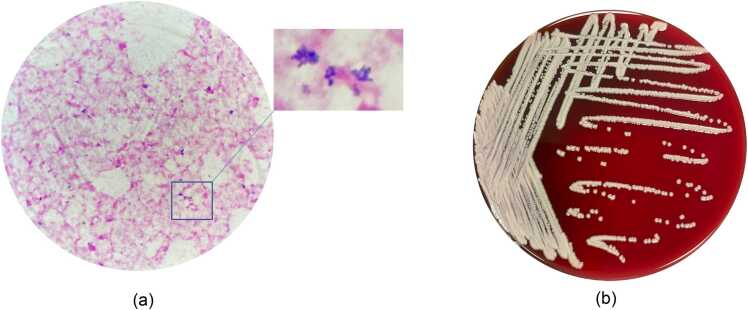
*B.ravenspurgense* morphology. (a) showed blood culture Gram staining (1000×) and (b) showed colony morphology on sheep blood agar.

**Fig. 2:**
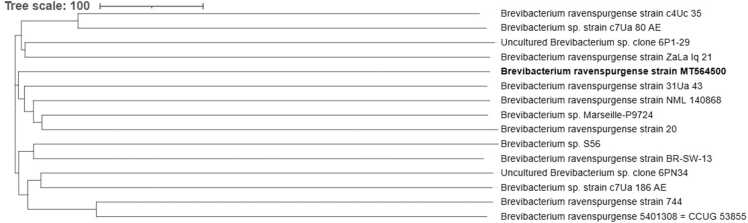
Phylogenetic tree based on 16S rRNA gene sequences.

Two sets of blood cultures were drawn to detect aerobic and anaerobic bacteria, and fungi (Becton Dickinson and Company, USA). One set was collected through a PICC line, and the other was obtained from a peripheral venipuncture. Instructions for Microbial culture vials indicate the aerobic vials can detect common clinical aerobic microorganisms such as *Haemophilus influenzae, Neisseria meningitidis*, *Streptococcus pneumoniae, Staphylococcus aureus, Pseudomonas aeruginosa*, and *fungi* such as *Candida albicans, Candida glabrata, and Cryptococcus neoformans*. The anaerobic vials can detect *Bacteroides fragilis, Clostridium histolyticum, Clostridium perfringens, Peptostreptococcus*, and *Veillonella parvula*. Additionally, we have performed performance verification using the above standard strains, achieving a 100 % positive coincidence rate. Both aerobic blood cultures turned positive within 48 h, revealing Gram-positive, club-shaped, slightly curved rods upon Gram staining (Fig. 1a). The antibiotic treatment was subsequently altered to intravenous tigecycline (50 mg q12h) for broader spectrum coverage. Following sub-culture on 5 % sheep blood agar, smooth, small colonies emerged after 48 h of incubation, accompanied by a distinct cheese-like odor (Fig. 1b). The isolate was further identified as *Gardnerella vaginalis* with a high degree of confidence using the commercially available PHOENIX M50 system (Becton Dickinson and Company, USA). However, *Gardnerella vaginalis* is a small, Gram-variable rod-shaped bacterium that does not produce hydrogen peroxide or catalase. It is one of the major pathogens causing bacterial vaginosis in women. Matrix-assisted laser desorption/ionization time-of-flight (MALDI-TOF) mass spectrometry was performed, but the isolate could not be identified using the VITEK MS system (bioMérieux, Marcy-l’Etoile, France). Genetic investigation by 16S rRNA analysis was then performed. Phylogenetic tree was constructed using the neighbor-joining method, included in the MEGA12 suite software,based on a comparison of approximately 1240 nucleotides. Bootstrap values,expressed as percentages of 1000 replications, are given at each branching point in the figures. Phylogenetic tree revealed that the isolate was *Brevibacterium ravenspurgense* strain MT564500 (Fig. 2).

Antimicrobial susceptibility testing was conducted using the broth microdilution methods, adhering to the Clinical and Laboratory Standards Institute guidelines for Corynebacteria. [5] The isolate was susceptible to vancomycin, meropenem, tetracycline, gentamicin, linezolid, but resistant to penicillin, cefepime, cefotaxime, ciprofloxacin, erythromycin, clindamycin, rifampicin, and tigecycline exhibited lower MIC as shown in Table 2.

**Table 2:** Antimicrobial susceptibility of *B.ravenspurgense* in the present study.

Antimicrobial agent	MIC (μg/mL)	Interpretation	Breakpoint(μg/mL)
Penicillin	8	R	≤ 0.12 ≥ 4
Vancomycin	0.125	S	≤ 2
Cefepime	4	R	≤ 1 ≥ 4
Cefotaxime	4	R	≤ 1 ≥ 4
Meropenem	0.125	S	≤ 0.25 ≥ 1
Ciprofloxacin	32	R	≤ 1
Gentamicin	4	S	≤ 4 ≥ 16
Erythromycin	4	R	≤ 0.5 ≥ 2
Clindamycin	4	R	≤ 0.5 ≥ 2
Tetracycline	2	S	≤ 4 ≥ 16
Rifampicin	4	R	≤ 1 ≥ 4
Linezolid	0.125	S	≤ 2
Tigecycline	0.25	NA*	NA*

MIC minimum inhibitory concentration; S susceptible; R resistant

* There are no agreed breakpoints for Tigecycline.

The patient's fever finally subsided, and baseline laboratory tests, including a complete blood count, inflammatory biomarkers, and blood cultures, were unremarkable following therapy lasting 7 days. The patient was discharged on the 60th day of hospital stay.

## Discussion

*Brevibacterium* species are nonmotile, catalase-positive, obligate aerobic, gram-positive rods associated with milk products and also found on human skin [6]. However, they have been reported as causative agents of human infection, including endocarditis, peritonitis, osteomyelitis, and bacteremia, particularly involving indwelling foreign material and in immunocompromised individuals [7], [8].

*B.ravenspurgense* is an emerging pathogen with limited clinical data available. To date, four research articles addressing *B.ravenspurgense* infections, including our own study, have been published [3], [9], [10], as summarized in Table 3. Among these studies, one was conducted in Germany, where the type strain was first isolated, two were from France, and one originated from China. The age range of patients varied widely, spanning from 4 to 88 years old, with a notable predominance of males who accounted for four out of five cases. The clinical manifestations associated with *B.ravenspurgense* infections differed based on the infection type. Patients suffering from wound infections typically presented symptoms characterized by exudate formation. In contrast, most individuals with bloodstream infections caused by *B.ravenspurgense* exhibited elevated body temperature along with moderately increased CRP levels. Furthermore, it has been suggested that immunocompromised states, such as those seen in patients with congenital metabolic disorders may increase susceptibility to bloodstream infections attributed to this organism. However, given the small sample size, partial data missing, and the long-time span of the study as well as the uneven geographical distribution, it is necessary to expand the sample size to verify the universality of the conclusions. Moreover, we noticed that one patient presented with a metabolic disorder known as methylmalonic acidemia. To date, however, no clear association between metabolic disorders and Brevibacterium ravenspurgense infections has been established. We anticipate that future research will uncover the underlying biological mechanisms behind this observation.

**Table 3:** Reports of *B.ravenspurgense* infections.

NO.	Year	Sex,age (y)	Species	Location	Underlying disease	Symptoms	Results	Device	Source	Identification	Drug susceptibility results	Treatment	Outcome
1	2008	M,45	ravenspurgense	Ravensburg, Germany	Not presented	Not presented	Not presented	Not presented	Wound swab	16S rRNA	Not presented	Not presented	Not presented
2	2008	F,88	ravenspurgense	Ravensburg, Germany	Not presented	Not presented	Not presented	Not presented	Blood	16S rRNA	Not presented	Not presented	Not presented
3	2009	M,78	massiliense	Marseille, France	NO	Discharge	Not presented	Not presented	Discharge	16S rRNA	Susceptible to rifampicin, doxycycline, vancomycin and amoxicillin, but was resistant to trimethoprim/sulfamethoxazole, erythromycin and gentamicin	Not presented	Not presented
4	2017	M, 4	massiliense	Marseille, France	MMA	Fever,cough and vomiting	WBC= 9.4*10^9/L, CRP = 0.59 mg/L	Gastrostomy tube	Blood	16S rRNA	Susceptible to amoxicillin, amoxicillin clavulanic acid, cefixime, imipenem, ofloxacin and vancomycin, but was resistant to trimethoprim/sulfamethoxazole, erythromycin and gentamicin	Cefixime + Ofloxacin	Improved
5	2023	M, 45	ravenspurgense	Renhuai,China	ALD	Fever	WBC= 12.53*10^9/L,CRP = 38.26 mg/L	PICC	Blood	16S rRNA	Susceptible to vancomycin,meropenem,tetracycline,gentamicin,linezolid, but resistant to penicillin,cefepime,cefotaxime,ciprofloxacin,erythromycin,clindamycin,rifampicin, and tigecycline exhibited lower MIC	Tigecycline	Improved

M Male; F Female; MMA methylmalonic acidemia; ALD Adrenoleukodystrophy

*B.ravenspurgense* recognition in routine clinical practice is limited because of it being a relatively newly described pathogen. In this case, misidentification initially occurred when the PHOENIX M50 system identified the isolate as *Gardnerella vaginalis*. According to the manual of PHOENIX M50 system, this device cannot identify Brevibacterium but can identify Gardnerella. As we understand, Brevibacterium grows relatively slowly and its biochemical reactions are not active enough. It is possible that some test results of Brevibacterium on this identification instrument are similar to those of Gardnerella, which leads to the inability to distinguish between these two bacteria. This misstep emphasizes the limitations of traditional biochemical identification systems in accurately detecting rare pathogens. MALDI - TOF also failed to identify the isolate, further highlighting the need for molecular methods like 16S rRNA gene sequencing for reliable pathogen identification. Molecular techniques not only provide accurate species-level identification but also enable the discovery of new or rare pathogens in clinical specimens, which is essential for appropriate treatment and infection control.

The patient's underlying condition of adrenoleukodystrophy likely played a role in the development of this rare infection. Adrenoleukodystrophy is a rare inherited metabolic disorder that can lead to immune system dysregulation and overall debilitation. The patient's long-term hospitalization, presence of a subclavian peripherally inserted central catheter (PICC), and pre-existing pneumonia are additional risk factors for bloodstream infections. The presence of a PICC line, although showing no visible signs of infection at the insertion site, could have served as a portal of entry for *B.ravenspurgense*. This emphasizes the importance of considering indwelling medical devices as potential sources of infection, especially in immunocompromised patients.

Regarding treatment, the antimicrobial susceptibility testing results were crucial in guiding therapy. Vancomycin was the preferred agent for treating infections caused by this pathogen. The resistance of *B.ravenspurgense* to commonly used antibiotics such as penicillin, cefepime, cefotaxime, ciprofloxacin, erythromycin, clindamycin, and rifampicin has significant implications. It not only limits treatment options but also highlights the need for continuous surveillance of antibiotic resistance patterns among rare pathogens. Although tigecycline has a low MIC, there are no interpretive standards available for this microorganism, rendering it not necessarily the optimal treatment option.

From a clinical perspective, this case serves as a reminder for clinicians to consider rare pathogens in patients with complex medical histories and multiple comorbidities. A high index of suspicion is required, especially when conventional treatment for common infections fails. Early recognition, accurate identification, and appropriate antibiotic therapy are essential for improving patient outcomes. Additionally, improving laboratory diagnostic methods to enhance the detection of rare pathogens in a timely and accurate manner should be a priority. This would not only benefit individual patients but also contribute to better infection control measures and public health surveillance.

## CRediT authorship contribution statement

**Hu Yonglin:** Writing – original draft, Data curation. **Daimin Xiao:** Writing – review & editing. **Ou Xingkun:** Writing – original draft, Software, Data curation. **xingyan Fang:** Writing – original draft, Funding acquisition, Formal analysis, Data curation.

## Ethical approval

Ethics committee approval and informed consent have been obtained.This case study has been conducted in accordance with the Declaration of Helsinki and approved by the local ethics committee of Kweichow Moutai Hospital.

## Funding

The authors declare that financial support was received for the research, authorship, and/or publication of this article. This work was partly supported by Kweichow Moutai Hospital Research and Talent Development Fund Project (MTyk2022-27).

## Author statement

All authors have seen and approved the manuscript being submitted, have contributed significantly to the work, attest to the validity and legitimacy of the data and its interpretation, and agree to its submission to the IDCases. We attest that the article is the Authors' original work, has not received prior publication and is not under consideration for publication elsewhere. On behalf of all Co-Authors, the corresponding Author shall bear full responsibility for the submission.

## Consent

Written informed consent was obtained from the patient for publication of this case report and the accompanying images.

## Declaration of Competing Interest

All authors confirm that there are no conflicts of interest to declare, including personal, financial, or other conflicts of interest that could bias the work reported in this manuscript.

## References

[1] Munshi A., Alsaedi A., Baloush S., Samarin K., Althaqafi A., Al-Amri A. (2022). Brevibacterium Luteolum bacteremia: a case report and literature review. IDCases.

[2] Eidensohn Y., Wei A., Sirkin M., Dever L.L. (2021). Brevibacteria tibial osteomyelitis. IDCases.

[3] Mages I.S., Frodl R., Bernard K.A., Funke G. (2008). Identities of Arthrobacter spp. and Arthrobacter-like bacteria encountered in human clinical specimens. J Clin Microbiol.

[4] Bernard K.A., Pacheco A.L., Burdz T., Wiebe D., Huynh C., Bonner C. (2016). Brevibacterium massiliense (Roux and Raoult 2009) is a later heterotypic synonym of Brevibacterium ravenspurgense (Mages, Frodl, Bernard and Funke 2009), using whole-genome sequence analysis as a comparative tool. Int J Syst Evol Microbiol.

[5] Benson C.E., Tatem L. (2021). Successful treatment of brevibacterium bacteremia solely with antimicrobial therapy. Cureus.

[6] Ovsthus K.K., Sjavik K., Lier T., Klingenberg C. (2021). Antibiotic therapy of an infant with a brevibacterium casei ventriculoperitoneal shunt infection. Pediatr Infect Dis J.

[7] Roy S., Garcha A.S., Patel S.S., Rahman E., Adapa S. (2022). Brevibacterium casei induced peritonitis in a patient undergoing continuous cycler peritoneal dialysis: case report and literature review. J Community Hosp Intern Med Perspect.

[8] Shweta F., Gurram P.R., O'Horo J.C., Khalil S. (2021). Brevibacterium species: an emerging opportunistic cause of bloodstream infections. Mayo Clin Proc.

[9] Vecten M., Gouriet F., Cano A., Raoult D. (2017). Brevibacterium massiliense bacteremia. IDCases.

[10] Roux V., Raoult D. (2009). Brevibacterium massiliense sp. nov., isolated from a human ankle discharge. Int J Syst Evol Microbiol.

